# Global market power dataset of the primary foods industry

**DOI:** 10.1016/j.dib.2024.110495

**Published:** 2024-05-08

**Authors:** Adrian Rodriguez del Valle, Esteban Fernández-Vázquez

**Affiliations:** University of Oviedo, Avda. Del Cristo s/n, 33006, Oviedo, Spain

**Keywords:** Agriculture, Hunting, Fishing, Market inefficiencies, Economic development

## Abstract

The study of market power has garnered interest from academia, policymakers, and industry in recent times since the publication of de Loecker et al. (2020). This paper introduced a novel methodology to estimate the markup, a proxy commonly used to denote market power. Using said methodology, they found that the markup has been increasing nearly continuously since the 1980′s. Rising markups have been connected to a myriad number of negative economic developments, yet most papers are constrained to study these effects on specific industries related to manufacturing and service. Furthermore, even though data exists for a considerable number of countries globally, the quality and reliability is reduced when examining low-income economies.^1^

To circumvent these problems, the authors have devised an alternate approach to calculate the markup, not by using firm-level data but by using macroeconomic data and an estimation procedure based on Generalised Maximum Entropy (GME). The methodology permits the estimation of markups for virtually every country in the world and a substantial number of industries.

The dataset provides estimates of the markups for 170 countries in the world for the so-called Primary Foods industry (comprising agriculture, hunting, fishing, and logging). It was calculated by aggregating two datasets: the EORA input-output tables and the UN FAO-Stat database. The merged dataset produced a panel from which the markup estimates were then calculated.

The publication potential of this dataset is very high, as no other source exists that captures this detail of information across countries with different income levels. The Primary Foods industry is also crucial for the development of poorer countries, as it often accounts for a large portion of their economy. This dataset opens up avenues of research finding ways to reduce the markup, thereby making economies more efficient and potentially improving the welfare of agents within the economy.

The usage of macro-data opens up additional avenues of research not available to micro-data, including measuring the impact of Global Value Chains (a form of globalization), institutional quality, and more on markups.

Specifications Table

**Table:** 

Subject	Economics, Econometrics and Finance/Macroeconomics
Specific subject area	The data falls within the field of industrial organisation and provides measures of market power, concretely the markup, of the Primary Foods Industry for 170 countries of the world.
Data format	Filtered data, displaying only the relevant variables required for analysis
Type of data	Table
Data collection	The data was collected by first merging two auxiliary datasets. The first one is the EORA input-output tables, or more concretely, the EORA26 Tables in basic prices. These tables fulfilled most of the requirements to calculate the markup, including measures of total output, labor remunerations, and intermediate inputs. EORA has information on 190 countries and regions for the years 1990 to 2015, with values being denoted in US dollars at current prices The second dataset is from the United Nations Food and Agriculture Organization Database (FAO-Stat). In particular, the stocks of capital were used in conjunction with the aforementioned variables from EORA. This dataset contains information on 188 territories or countries for 1995 to 2015, although it contains missing information for several years. The stocks of capital are also valued in US Dollars at current prices. Both sources of data are accessible for free; the former requires only registering to the site. As the industrial classification between sectors differs between both datasets, two industries within EORA were merged using an identity matrix. This procedure and the estimation of the markup self, is explained in more detail in the paper by [6].
Data source location	The EORA input-output database can be accessed here: https://worldmrio.com/, requiring registration to verify the users’ academic credentials. The latter can be accessed through this link for free: https://www.fao.org/faostat/en/#data/CS
Data accessibility	Repository name: Mendeley Data Data identification number: 10.17632/879zmf9tzh.1 Direct URL to data: https://data.mendeley.com/datasets/879zmf9tzh/1
Related research article	Rodriguez del Valle and Fernández-Vázquez (2024)

## 1 Value of the Data

• This topic is of high political relevance. Unfortunately, there are severe constraints in calculating the markup because of the unavailability of firm-level micro-data. Most studies focus on developed countries with more advanced data collection systems. We circumvent this problem by calculating the markup using specialized, industry-level macro data, being the first to do so. Whereas firm-level (micro) studies link the markups with firm-level characteristics to derive correlations and causal relationships, macro-data opens up other channels that explain the markups.
• The authors have already found a significant relationship between globalization and markup, concluding that opening up to trade might be beneficial to the economy by reducing markups. However, there are considerable further avenues that are available. For example, evidence exists that institutional quality, or how efficient the Government is at creating sound policies and implementing them, can impact markups and economic efficiency. No exhaustive study has been done on this to date, however the dataset might allow us to do so.

## 2 Background

**Fig. 1:**
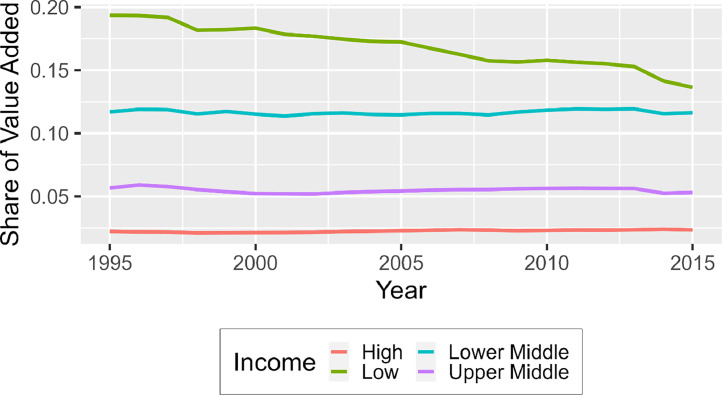
Share of value added of the primary foods industry relative to total value added, divided by income groups. *Note:* Aggregated using weighted averages based on value added. For ease of interpretation, only the income level classification for of 1995 was used for all countries. The calculations are based on the authors using data from the EORA Input-output Data.

Evidence exists that market power has been increasing since the 1980′s. The development is concerning since rising market power is connected with detrimental economic developments such as rising prices, decreasing output, decreasing innovation, and higher costs of entry into the market. These inefficiencies are particularly problematic for poorer countries, as the Primary Foods industry comprises a large part of their total economy. The dependency of low-income economies on the Primary Foods industry is visualized more clearly in Fig. 1, with higher income levels having progressively a lower share of value added for this industry.

The majority of studies to date analyzing the impact of market power do so using micro-level (firm-level) data, that is available for only high-income countries. The authors were motivated to propose an alternate way of deriving estimates of the markup to study this phenomenon for countries all over the world.

**Fig. 2:**
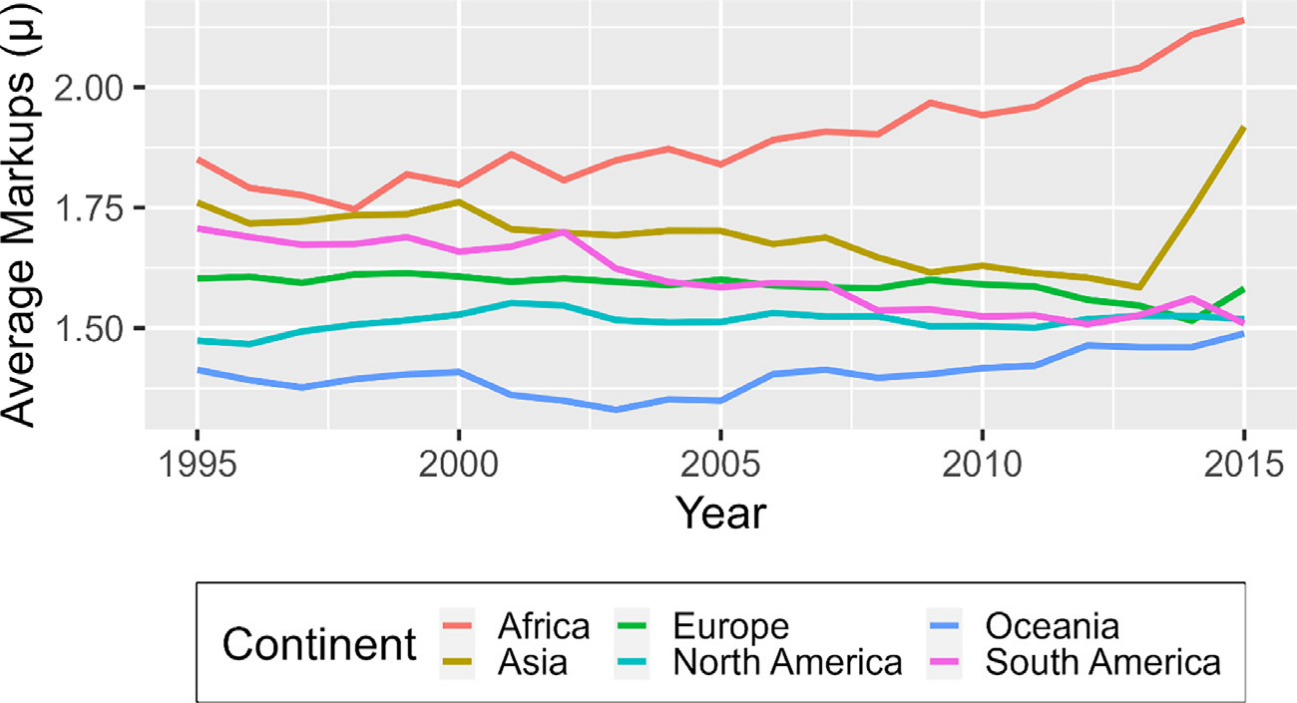
Evolution of Average Markups of the Primary Foods Industry, divided by Continent Note: Aggregated using weighted averages based on value added. The calculations are based authors using the database described in this paper.

High-income countries often share similar characteristics, such as having similar institutions, geographic areas, political systems, history, climate, and access to trade, to name a few. Current research, therefore, cannot easily discern the impact of these factors on markups. The data proposed here provides a wide enough coverage of countries so that research into many new topics is feasible, thereby potentially opening up newer avenues of research. Fig. 2 further reinforces this idea, as the evolution of markups for the Primary Foods Industry is relatively stable for Europe and North America, regions having predominantly high-income economies but more volatile for Africa and Asia. Noteworthy is that Africa is found to have a near-continuously increasing markup throughout the sample period between 1995 and 2015. The motivation to understand this discrepancy is therefore significant.

## 3 Data Description

The data is comprised of one single Excel file called “Global Markups Primary Food”. The file has several variables, including year, country, country code in ISO3 format, continent, and a subregion defined as “Region 1”. The variable “income” represents the World Bank's income classification for each country, categorized into four labels: “L” for low income, “LM” for lower medium income, “UM” for upper medium income and “H” for high income. The classification is dependent on the estimated Gross National Index per capita of each country using predefined ranges set by the World Bank for a specific year.

The database is constructed using two external datasets: the EORA input-output tables and the UN FAO-stat Database. A considerable number of variables present in the database were obtained using the EORA26 tables. These tables are simplified versions that contain information for only 26 industries, in contrast to the EORA full tables. The database makes use of the simplified prices, denoting values in current US Dollars. The authors extracted variable “L” directly from vector “Labor Remunerations” that is present in the EORA26 “bp_VA” files, the value-added matrix. The intermediate inputs vector “II” was calculated by summing the columns of the transaction matrix “bp_T”. Total output “Q”, was calculated by adding the sum of columns contained within the transaction matrix and the value added matrix. The variable VA represents the value-added vector and was calculated by subtracting Q (total output) from II (intermediate inputs). Variable K represents the value in current US Dollars of the stocks of capital for agriculture, hunting, logging, and fishing (defined here as the Primary Foods Industry). The industrial coverage from the FAO-stat is broader than the one from EORA, as it includes industries within ISIC Rev. 3 sectors A and B. Because of this, two industries from EORA were aggregated using an aggregation matrix. Concretely, industry “agriculture” comprising of agriculture, hunting, and logging (ISIC Rev. 3 sector 1 and 2, or A) was aggregated with industry “fishing” (ISIC Rev. 3 sector 5, or B) to make both datasets harmonious.

The aforementioned variables were then used to calculate the main highlights of the dataset: (i) the variable “markup“ describes the markup (μ) calculated using the GME method; (ii) the variable “rts” contains the estimated returns to scale calculated as the sum of the elasticities of output to variable inputs and output to capital (α and β respectively in the production function); and (iii) the variable “profit rate” describes the aggregate profits in relative terms to the gross revenues, based in the relationship rts=μ×(1−profitrate). The remaining variables are derived directly from the input-output tables. By order of appearance; Q represents total output, II are the intermediate inputs, VA is the value added, LAB is the total labor remunerations, and K is the stocks of capital. Subsidies are the subsidies given to the Primary Foods industry, and Taxes are the taxes obtained from the Primary Foods industry (Table 1, Table 2).

**Table 1:** Summary statistics markup by year.

Variable	Year	n	Min	Max	$\overset{‾}{x}$	s
Markup	1995	146	0.89	4.69	1.95	0.69
	1996	146	0.92	4.17	1.90	0.60
	1997	146	0.86	3.60	1.83	0.51
	1998	146	0.88	3.67	1.80	0.48
	1999	146	0.78	4.21	1.83	0.49
	2000	169	0.86	3.72	1.73	0.40
	2001	170	0.96	4.04	1.80	0.47
	2002	169	0.96	3.42	1.77	0.44
	2003	170	0.95	4.62	1.83	0.54
	2004	170	0.97	3.93	1.86	0.57
	2005	169	0.96	3.81	1.85	0.54
	2006	170	0.95	4.05	1.89	0.59
	2007	170	0.96	4.20	1.90	0.60
	2008	169	0.95	4.05	1.89	0.59
	2009	169	0.92	4.38	1.92	0.64
	2010	169	0.89	4.37	1.92	0.64
	2011	167	1.03	4.35	1.93	0.65
	2012	168	0.90	5.24	2.00	0.75
	2013	168	0.89	6.06	2.04	0.82
	2014	169	0.89	6.94	2.07	0.90
	2015	167	0.92	6.01	2.05	0.82
	all	3433	0.78	6.94	1.89	0.63

**Table 2:** Summary statistics markup by continent.

Variable	Continent	n	Min	Max	$\overset{‾}{x}$	s
Markup (μ)	Africa	988	0.78	6.94	2.11	0.74
	Asia	862	0.89	4.62	1.77	0.55
	Europe	792	0.89	4.98	1.61	0.35
	North America	421	0.99	5.07	1.99	0.67
	Oceania	147	1.57	4.60	2.33	0.77
	South America	223	1.07	3.19	1.92	0.34
	all	3433	0.78	6.94	1.89	0.63

The data contains 82 missing values, stemming from the lack of information on the stocks of capital for some countries.

## 4 Experimental Design, Materials and Methods

The point of departure for deriving markups is based on the works by De Loecker and Eeckhout [2] and De Loecker et al. [3]. The authors exploit rich databases at firm level to estimate Cobb-Douglas production functions with variable (intermediate consumptions and labour) and fixed inputs (capital stock) as:

(1) $$Y_{i}\;=\Omega \;V_{i}^{\alpha }K_{i}^{\beta }$$

Where $Y_{i}$ stands for the output, $V_{i}$ and $K_{i}$ represent the variable inputs and the stock of capital respectively, and Ω is a measure of the factor productivity for a company i. Let us denote the mark-up of company ($\mu_{i}$) It can be proved that, after some manipulations, $\mu_{i}$ can be calculated as follows:

(2) $$\mu_{i}=\alpha \frac{Y_{i}}{V_{i}}$$

The application of this expression simplifies the estimation of mark-ups, since the elasticities α can be retrieved from the estimates of econometric regressions of the production functions like (1), while $Y_{i}$ and $V_{i}$ can be observed directly in some datasets. Actually, De Loecker and Eeckhout [2] follow this approach to study the evolution of market power approximated by the evolution of mark-ups from a database containing detailed information at company level. While this strategy is appealing when dealing with specific industries (manufactures and services) and countries (i.e., western economies) covered in these datasets, it cannot be directly applied to the study of market power for agricultural activities in non-western countries.

There have been recent attempts to overcome these difficulties by, following the idea of Hall [4] of studying market power with aggregated data, apply a similar strategy to macro-indicators present in global input-output (IO) databases. Colonescu [1] and Rodriguez del Valle and Fernandez-Vazquez [5], take advantage of the information contained in the World Input-Output database (WIOD) to estimate nark-ups for European manufacturing industries in recent years. Similarly, the strategy followed in this paper is to use data comprised in global IO database and other global datasets with a similar purpose for the agricultural activities of a set of 43 Asian countries. More specifically, we use the information on output ($Y_{i}$) and variable inputs ($V_{i}$) observable on a yearly basis in the EORA database for the agricultural sector between 1995 and 2015 for the countries under study. Additionally, we will complement our required variables with data of capital stocks ($K_{i}$) for the agricultural industries in these countries coming from the FAO database.^2^

With these data at hand, we estimate equations like (1) for each year between 1995 and 2015. In our analysis, i does not refer to a company, but it stands for the agricultural sector in one country. We assume that elasticities are common within continents but evolving along time. This implies that we need to estimate a production function for each continent and year. Our approach requires, in consequence, estimating a total of 21 years × 6 continents regressions. We follow this approach to accommodate the larger flexibility possible by allowing time-varying coefficient in the production functions. The cost for this flexibility is the reduction in the number of data points on each regression, and the GME estimator is particularly attractive in such ill-conditioned samples where traditional estimation techniques relying on larger sample sizes can be problematic. We follow the same strategy as in Rodriguez del Valle and Fernandez-Vazquez (2023) and apply a Generalized Maximum Entropy (GME) estimator. This estimator has the advantage of producing robust estimates with limited data by means of a reparametrization of the elasticities of interest and the error term.

The GME estimator reparametrizes the element of a typical linear regression y=Xβ+u in terms of probability distributions. Each element of the vector of parameters β is assumed to be a discrete random variable with M≥2 possible realizations. These potential values of the unknown parameter are included in a support vector $b{}_{h}^{\prime }=b_{h1},...,b_{hM}$ with corresponding –-unknown– probabilities $p{}_{h}^{\prime }=\left(p_{h1},...,p_{hM}\right)$. A similar approach is followed for the random disturbances. Although GME does not require specific assumptions about the probability distribution function of the noise term, some assumptions are necessary. First, the uncertainty about the realizations of this element is addressed by treating each element $u_{i}$ as a discrete random variable with J≥2 possible outcomes contained in a convex set $v{}^{\prime }=v_{1},...,v_{J}$ which, for the sake of simplicity, will be common for all the realizations of the random disturbance $u_{t}$. Second, we also assume that these possible outcomes of the random disturbance are symmetric and centered on zero ($-v_{1}=v_{J}$). As a result, u has mean E[u]=0 and a finite covariance matrix Σ. Additionally, it is common practice to establish the upper and lower limits of the vector v applying the three-sigma rule (±3 times the standard deviations of the dependent variable in the sample). Under these conditions and some mild assumptions, GME estimates distribute as $\hat{\beta }\to N\left[\beta ,{\hat{\sigma }}^{2}{\left(X^{\prime }X\right)}^{-1}\right]$ and it is possible to obtain their approximate variance matrix as ${\hat{\sigma }}^{2}{\left(X^{\prime }X\right)}^{-1}$.

Our application of the GME estimator requires the specification of supporting vectors for the parameters and the error terms. The parameters be estimated are the output elasticities $\alpha_{it}$ and $\beta_{it}$ and the factor productivities $\Omega_{it}$. For the term $\Omega_{it}$ we set support vectors with M=3values ($b_{\Omega m}$) centered at 0 and with bounds at ±2. For the output elasticities we define supporting vectors with M=3points ($b_{\alpha m}$ and $b_{\beta m}\;$respectively) centered at the corresponding mean value of the shares of variable inputs and the stock of capital, being the limits of these vectors set as these means plus and minus 2 again, in order to assure having wide enough supports. This approach implies that, in absence of information, the GME estimator produces uniform probabilities, and the point estimates of the parameters will be equal to the central value in the vectors. Consequently, the uninformative GME solution makes the mean markup $\mu_{itc}$ equal to one by construction. In other words, our prior assumption is that there is no market power and only if data contains information that contradicts this initial assumption, the GME estimator will produce a different result

## Limitations

One limitation of the dataset is the assumption the authors had to take for the values to be computable. The assumption is that each industry is produced by one representative firm, as explained in more detail in Rodriguez del Valle and Fernández Vázquez (2023; 2024). The assumption is most likely not critical in countries having farms of similar size, as this assumption would represent the markup of an average farm. However, the assumption might be more critical when the industry is comprised of a few large firms that dominate the market. In such cases, the results might be biased. In contrast, relying on aggregate data instead of exploiting individual firm observations allows us to limit the “transmission bias” problem, which frequently appears when firm-specific productivity shocks impact the use of inputs, biasing the estimates of elasticities by traditional econometric techniques. However, aggregated, industry-level, shocks might still be a concern.

Large farm sizes appear in high-income countries and are nearly non-existent in low-income countries. As such, caution is needed when examining this industry for high-income countries. Furthermore, the macrodata contained within the EORA input-output tables will occasionally estimate values for the industries of certain countries when no data is present. In these cases, a certain bias might also arise. Similarly, for some continents and years the datapoints used to conduct the estimates are reduced, and our estimates are based in these small datasets. Additionally, the estimates presented in the database are naturally limited by the validity of the assumptions made to model the production technology, in particular the assumption of a common production function for each continent and year.

## Ethics Statement

The authors confirm having read Data in Brief's ethical requirements. The authors have followed these requirements closely while producing the dataset to ensure that ethical standards were upheld. Furthermore, the creation of the dataset did not involve human subjects, animal experiments and the use of social media.

## CRediT authorship contribution statement

**Adrian Rodriguez del Valle:** Conceptualization, Data curation, Validation, Writing – original draft. **Esteban Fernández-Vázquez:** Methodology, Software, Writing – review & editing.

## Data Availability

Global Markup Estimates for the Primary Foods Industry (Original data) (Mendeley Data).

## Footnotes

2 Details on the datasets employed are presented in the section with the data description.
